# Mr. Davie, on Ague

**Published:** 1804-12-01

**Authors:** L. Davie

**Affiliations:** Framlingham, Suffolk


### *To the Editors of the Medical and Physical Journal.*

GENTLEMEN,

I Have read with much pleasure, in your highly useful Journal of the last month, an ingenious and liberal communication on the cure of agues, by your correspondent Mr. Snow. I cannot but think it reflects the highest credit on him and all others, who come forward with so much professional candour to state facts, unbiassed by prejudice or false hypothetical reasoning and theory, or making real truth and actual benefit, as many do, their last consideration. I am most decidedly an advocate for the use of emetics in the cure of agues, in the form of nauseating doses, as has been recommended. You certainly act by them on the skin in a way that cannot be done by any other means, stimulating the excretory surface, relaxing its spasm, cutting short the rigour, altering its form, and ultimately curing this kind of fever; for although I do not consider spasm as the cause of intermittent fever, but as one of the train of symptoms, yet no doubt its removal by this stimulus on the sentient extremities of the nerves, is always importantly necessary to the cure. My object, then, in this, is to suggest an improvement in the emetics had recourse to in the cases mentioned by Mr. Snow; it is what I, in several cases of late, have uniformly used, and in most instances with the happiest effect. I unite with the tart. antim. an equal part of the cuprum vitriol. and two parts of the pulv. ipecac.; this I give in small and repeated doses, so as to produce nausea or slight vomiting at the commencement of each paroxysm. The advantage I think of this medicine over that of the tart. antim. alone is, that with its emetic property is combined a tonic one. The repeated use of it does not interfere with or lessen the tone of the stomach; the strength of the system is kept up and increased, Nature is enabled to exert herself, and, with this assistance of art, to throw off the morbid impression made upon her by the poison of fever. I am, &c.

*Framlingham, Suffolk, Oct.* 14, 1804. L. DAVIE.

